# Power analysis for RNA-Seq differential expression studies using generalized linear mixed effects models

**DOI:** 10.1186/s12859-020-3541-7

**Published:** 2020-05-19

**Authors:** Lianbo Yu, Soledad Fernandez, Guy Brock

**Affiliations:** Center for Biostatistics, Department of Biomedical Informatics, The Ohio State University, 1800 Cannon Dr., Columbus, 43210 OH USA

**Keywords:** RNA-Seq, Power analysis, Bivariate negative binomial, Generalized linear mixed effects Model

## Abstract

**Background:**

Power analysis becomes an inevitable step in experimental design of current biomedical research. Complex designs allowing diverse correlation structures are commonly used in RNA-Seq experiments. However, the field currently lacks statistical methods to calculate sample size and estimate power for RNA-Seq differential expression studies using such designs. To fill the gap, simulation based methods have a great advantage by providing numerical solutions, since theoretical distributions of test statistics are typically unavailable for such designs.

**Results:**

In this paper, we propose a novel simulation based procedure for power estimation of differential expression with the employment of generalized linear mixed effects models for correlated expression data. We also propose a new procedure for power estimation of differential expression with the use of a bivariate negative binomial distribution for paired designs. We compare the performance of both the likelihood ratio test and Wald test under a variety of simulation scenarios with the proposed procedures. The simulated distribution was used to estimate the null distribution of test statistics in order to achieve the desired false positive control and was compared to the asymptotic Chi-square distribution. In addition, we applied the procedure for paired designs to the TCGA breast cancer data set.

**Conclusions:**

In summary, we provide a framework for power estimation of RNA-Seq differential expression under complex experimental designs. Simulation results demonstrate that both the proposed procedures properly control the false positive rate at the nominal level.

## Background

RNA-Seq has become a popular tool for studying dynamics of gene function through transcriptomic data of individuals with multiple samples of different origins [1, 2], diverse cell types [3, 4], and multiple time points [5, 6] over the past decade. The transcriptomic measurements from multiple samples of the same individual are correlated in nature. It is very challenging to estimate power in designing these RNA-Seq experiments as well as to do comparative analysis. Currently there is a need of developing statistical methods for sample size calculation and power estimation with correlated RNA-Seq data.

Since the emergence of RNA-Seq data, several papers have used Poisson or negative binomial (NB) distribution to model count-based expression data [7–9]. But these methods are based on generalized linear models with fixed effects, so they can not be directly applied to correlated expression data. To overcome this limitation, others proposed the generalized linear mixed effects model (GLMM) to model count-based expression data by adding random effects to allow diverse correlation structures while still assuming a Poisson distribution (Poisson-LMM) or NB distribution (NB-LMM) [10–12]. In addition, the bivariate negative binomial (BNB) distribution was introduced to model paired counts of brain lesions [13]. The BNB distribution is a compound distribution of two conditionally independent Poisson random variables for modeling paired counts with a Gamma random variable for modeling individual effects. Even though the BNB distribution has not yet been used for RNA-Seq data analysis, it is a great candidate for experiments using paired designs.

Several studies provide methods for sample size calculation and power estimation at the marginal level [14–17] or data set level [18–20] for testing differential expression of RNA-Seq experiments. However these methods were designed for experiments using independent samples and can not be directly applied to correlated expression data since they may lead to biased estimators of model parameters and result in a failure of proper error rate control. So far, there is lack of general methods for power analysis that can be applied to correlated RNA-Seq data. To overcome this deficiency, we are the first group to propose a BNB approach for paired designs and a more general GLMM approach for designs with diverse correlation structures. To ensure the false positive rate is properly controlled at the nominal level, we employ our previously published procedure for simulating the null distribution of test statistics [17] and also compare it against the asymptotic Chi-square distribution. To demonstrate performance of the new BNB and GLMM approaches, simulations were conducted under a variety of scenarios. A real TCGA data set was used for method application.

## Methods

### BNB model

The BNB distribution can be obtained by compounding two conditionally independent Poisson random variables *X*|*G*=*g*∼*Poisson*(*μg*) and *Y*|*G*=*g*∼*Poisson*(*γμg*) with a Gamma random variable *G*∼*Gamma*(*ϕ*^−1^,*ϕ*). The probability mass function for the joint distribution of (*X*,*Y*) is

$$\begin{array}{@{}rcl@{}} P(X=x,Y=y) = \frac{\phi^{-\phi^{-1}}}{\Gamma\left(\phi^{-1}\right)} \frac{\mu^{x}\left(\gamma\mu \right)^{y}}{\Gamma(x+1)\Gamma(y+1)} \frac{\Gamma\left(x+y+\phi^{-1}\right)}{\left(\mu+\gamma\mu+\phi^{-1}\right)^{x+y+\phi^{-1}}}. \end{array} $$

Without loss of generality, we use *γ* to denote the fold ratio of a gene between two conditions. We are interested in testing hypothesis *H*_0_:*γ*=*γ*_0_ vs. hypothesis *H*_1_:*γ*≠*γ*_0_. A Wald test for testing log transformed *γ* is *H*_0_:*log*(*γ*)=*log*(*γ*_0_) vs. *H*_1_:*log*(*γ*)≠*log*(*γ*_0_). The likelihood ratio test (LRT) statistic and the Wald test statistic for the above hypothesis with BNB distribution are defined in Rettiganti and Nagaraja [13].

### GLMM

Poisson or NB distribution is modeled through the log link function of a linear predictor of mixed effects as

$$\begin{array}{@{}rcl@{}} \eta = X\beta + Zb, \end{array} $$

where *β* are fixed effects and *b* are random effects following normal distributions. For inference on fixed-effects *β*, hypotheses *H*_0_:*Lβ*=0 vs.*H*_1_:*Lβ*≠0 is tested by LRT or Wald test. Random effects *b* can be tested by z-statistic for difference from 0.

### Empirical parametric test

In this study, we used our previously published simulation-based empirical parametric test for inferences [17] and the extended Bonferroni method for controlling per comparison error rate (PCER) [21].

### Procedure for power estimation

1. Specify all input parameters: sample size per condition *n*; mean expression *μ*; dispersion *ϕ*; fold ratio *γ* between conditions, nominal false positive rate *α*, number of simulations *T*.
2. Simulate count data *T* times from BNB(*μ*,*γ*,*ϕ*) under both the null and alternative hypotheses using the input parameters listed in Step 1.
3. Fit BNB model or GLMM and obtain test statistics (LRT or Wald) under the null hypothesis for each simulation run.
4. Calculate the 100(1−*α*)th percentile of the empirical null distribution of test statistics (LRT or Wald) as the critical value.
5. Fit BNB model or GLMM and obtain test statistics (LRT or Wald) under the alternative hypothesis for each simulation run.
6. Calculate power (percent of rejections under the alternative hypothesis) for the input parameters listed in Step 1.

## Results

### Simulations

#### Parameter setting

Count data were simulated from a Poisson-Gamma (BNB) distribution under two experimental conditions (e.g. baseline vs. treatment) for *n* subjects. The input parameters for power calculation at the marginal level are sample size *n*, mean expression *μ* at baseline, fold ratio *γ* between the two conditions, dispersion *ϕ*, and nominal false positive rate *α*. Parameter values for each are $n=5, 10, 15, 20, 25; \mu =3, 5, 10, 20, 100; \gamma =\frac {1}{3},\frac {1}{2},1,2,3; \phi =0.01, 0.1, 1, 10, 100; \alpha =0.01, 0.005, 0.001, 0.0005$. Under Poisson-LMM and NB-LMM models, experimental condition is the fixed effect and subject is the random effect. Under each scenario, 20,000 simulations were run as described in section ‘Procedure for Power Estimation’.

#### Power analysis

**Fig. 1:**
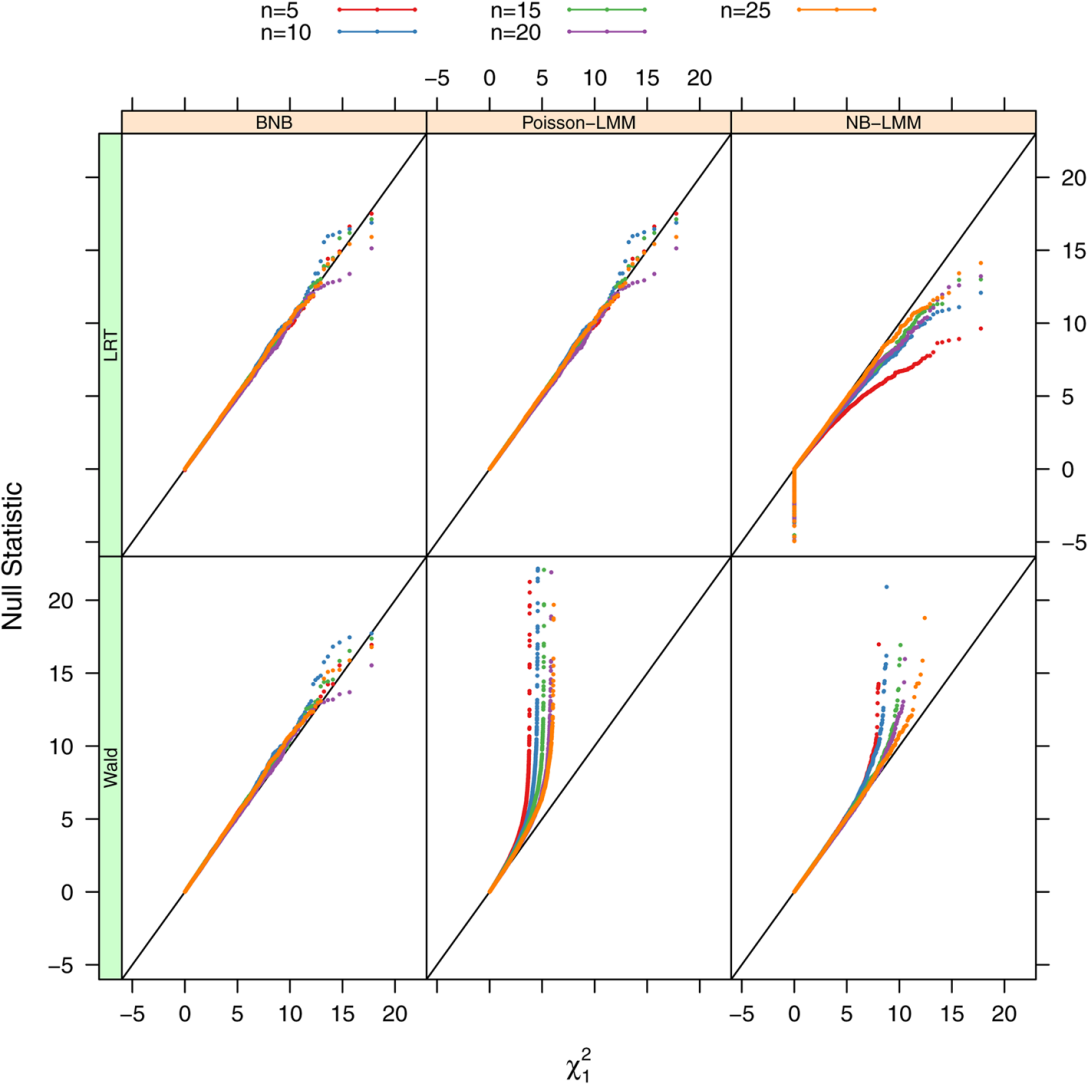
QQ plots of null LRT and Wald statistics. Data were simulated for 20,000 times at *μ*=10 and *ϕ*=1 under the null hypothesis. Sample sizes were set at *n*=5,10,15,20,and 25. Both the LRT and Wald tests were used for testing mean differences between two conditions under the BNB, Poisson-LMM, or NB-LMM model. Null test statistics are plotted against the Chi-square distribution with 1 degree of freedom. Both the LRT under the BNB model and the LRT under the Poisson-LMM model are approximately following the Chi-square distribution. The Wald test under BNB is slightly above the Chi-square distribution. The LRT under the NB-LMM model is moderately below the Chi-square distribution. Both the Wald test under the Poisson-LMM model and the Wald test under the NB-LMM model are dramatically above the Chi-square distribution

Figure 1 shows QQ plots for both LRT and Wald test statistics under the BNB, Poisson-LMM, and NB-LMM models at the null hypothesis with *n*=5,10,15,20,25; *μ*=10; *ϕ*=1. The null distribution of LRT statistics under the BNB model can be approximated to a Chi-square distribution with 1 degree of freedom, but Wald test statistics under the BNB model are slightly above the Chi-square distribution. The null distribution of LRT statistics under the Poisson-LMM model can be approximated to a Chi-square distribution with 1 degree of freedom, but LRT statistics under the NB-LMM model are moderately below the Chi-square distribution. Wald test statistics under both the Poisson-LMM and NB-LMM models have unusually large values due to computational instability and dramatically deviate from the Chi-square distribution, therefore these two tests are not pursued further for power analysis.

**Fig. 2:**
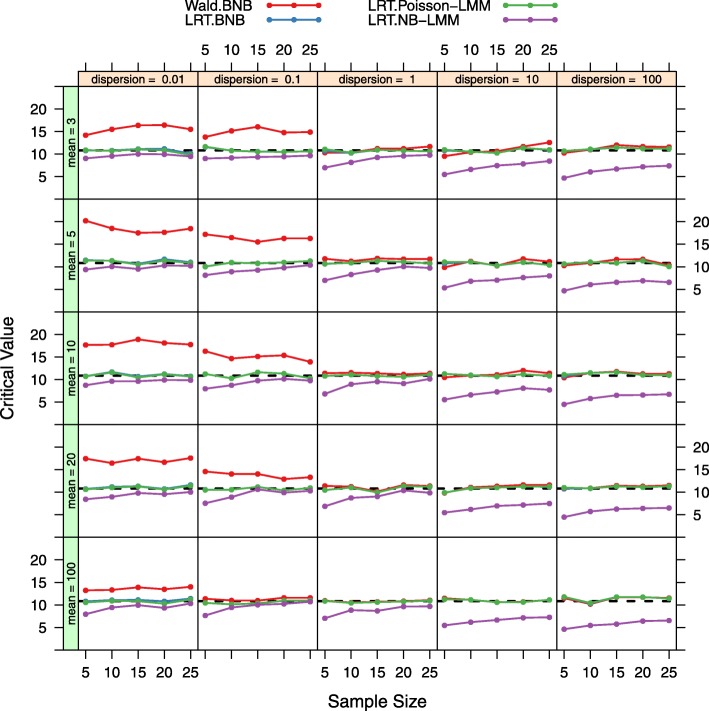
Critical value plots for both the LRT and Wald tests. Critical values were calculated from the empirical null statistics at 5 different sample sizes, 5 different mean expression levels, 5 different dispersion values for four tests (LRT under the BNB model, Wald test under the BNB model, LRT under the Poisson-LMM model, LRT under the NB-LMM model) and for the Chi-square distribution with 1 degree of freedom (dashed black lines). The nominal false positive rate *α* is set at 0.001. The LRT under the BNB model and the LRT under the Poisson-LMM model have similar critical values as the Chi-square distribution, but the Wald test under the BNB model has larger critical values than the Chi-square distribution especially at smaller mean expression levels and oppositely the LRT under the NB-LMM model has lower critical values

**Fig. 3:**
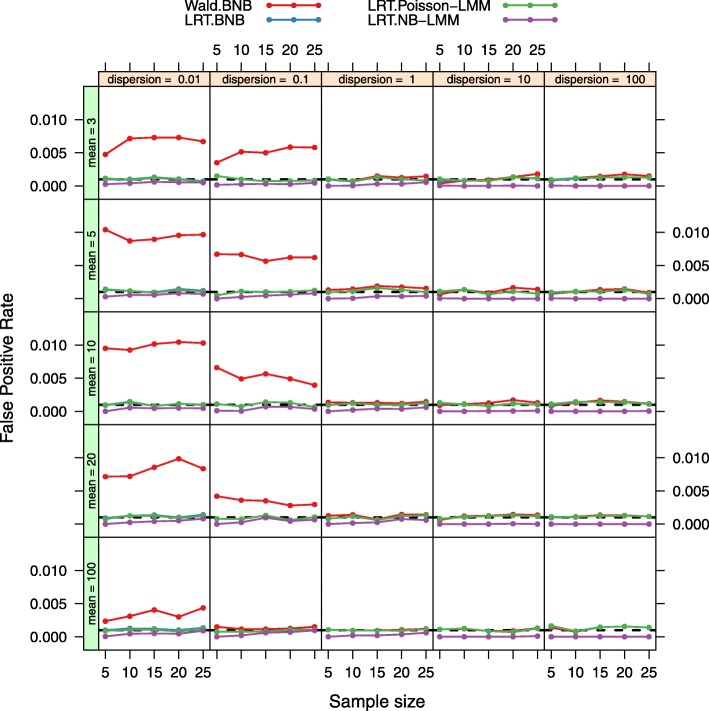
False positive rate plots for both the LRT and Wald tests. Actual false positive rates were calculated at 5 different sample sizes, 5 different mean expression levels, 5 different dispersion values for four tests (LRT under the BNB model, Wald test under the BNB model, LRT under the Poisson-LMM model, LRT under the NB-LMM model). The nominal false positive rate *α* is set at 0.001 (dashed black lines). The Asymptotic Chi-square distribution is used instead of the empirical parametric test for calculating the critical values. Actual false positive rates of the LRT under the BNB model and the LRT under the Poisson-LMM model are well maintained at the nominal false positive rate. Actual false positive rates of the Wald test under the BNB model are much larger than the nominal false positive rate especially at smaller mean expression levels. Actual false positive rates of the LRT under the NB-LMM model are uniformly lower than the nominal false positive rate

Figure 2 shows the critical values of the empirical null distribution for the four tests (LRT under the BNB model, Wald under the BNB model, LRT under the Poisson-LMM model, and LRT under the NB-LMM model) and the critical values of the Chi-square distribution with 1 degree of freedom over 5 different sample sizes, 5 different mean expression levels, and 5 different dispersion parameters. At each input parameter setting, the false positive rate is controlled at the nominal level *α*=0.001 by using the empirical parametric test or the Chi-square distribution. Both the LRT under the BNB model and the LRT under the Poisson-LMM model with the use of the empirical parametric test have similar critical values as the Chi-square distribution. However the Wald test under the BNB model with the use of the empirical parametric test has larger critical values than the Chi-square distribution especially at smaller mean expression levels. The LRT under the NB-LMM model with the use of the empirical parametric test has lower critical values than the Chi-square distribution. When the approximated Chi-square distribution is used instead of the empirical parametric test (Fig. 3), actual false positive rates of the LRT under the BNB model and the LRT under the Poisson-LMM model are well maintained at the nominal false positive rate as expected. Actual false positive rates of the Wald test under the BNB model are much larger than the nominal false positive rate especially at smaller mean expression levels. Actual false positive rates of the LRT under the NB-LMM model are uniformly lower than the nominal false positive rate.

**Fig. 4:**
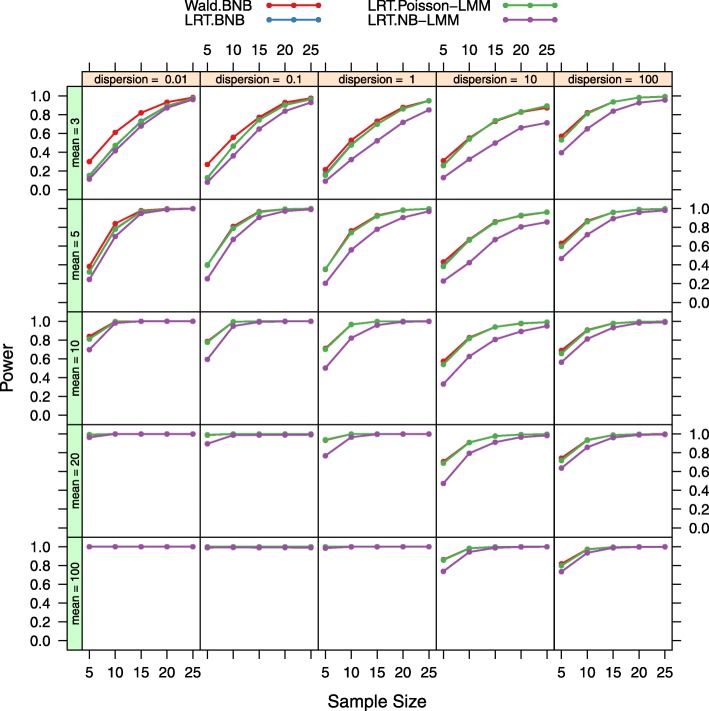
Power plots for both the LRT and Wald tests at the fold ratio of 2. Power was calculated at 5 different sample sizes, 5 different mean expression levels, 5 different dispersion values for four tests (LRT under the BNB model, Wald test under the BNB model, LRT under the Poisson-LMM model, LRT under the NB-LMM model) of the fold ratio of 2. The nominal false positive rate *α* is set at 0.001. The LRT under the BNB model and the LRT under the Poisson-LMM model have equivalent power over different parameter values. But compared to them, the Wald test under the BNB model has higher power and the LRT under the NB-LMM model has lower power at almost all parameter values

**Fig. 5:**
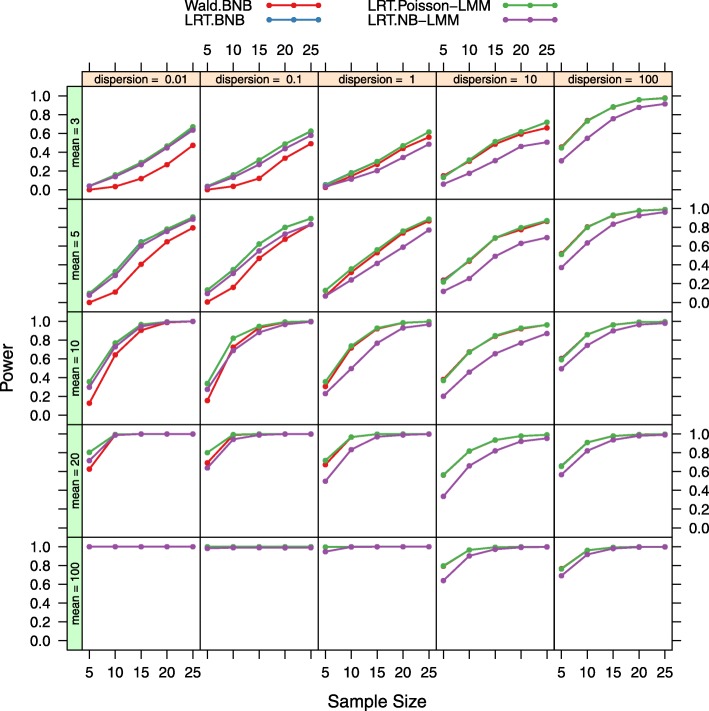
Power plots for both the LRT and Wald tests at the fold ratio of 0.5. Power was calculated at 5 different sample sizes, 5 different mean expression levels, 5 different dispersion values for four tests (LRT under the BNB model, Wald test under the BNB model, LRT under the Poisson-LMM model, LRT under the NB-LMM model) of the fold ratio of 0.5. The nominal false positive rate *α* is set at 0.001. The LRT under the BNB model and the LRT under the Poisson-LMM model have equivalent power over different parameter values. But compared to them, the Wald test under the BNB model has lower power and the LRT under the NB-LMM model has lower power at almost all parameter values

Power at two different fold ratios (2 fold down or 2 fold up respectively) for the four tests at *α*=0.001 are displayed in Figs. 4 and 5. In general, power increases with larger sample sizes, larger mean expression levels, and larger absolute fold changes for all four tests. The LRT under the BNB model and the LRT under the Poisson-LMM model have equivalent power over different parameter values. The Wald test under the BNB model has lower power at 2 fold down and higher power at 2 fold up in general when compared to the LRT under the BNB model and the LRT under the Poisson-LMM model. The LRT under the NB-LMM model has lower power at almost all parameter values when compared to the LRT under the BNB model and the LRT under the Poisson-LMM model.

### Applications

#### TCGA data set

**Fig. 6:**
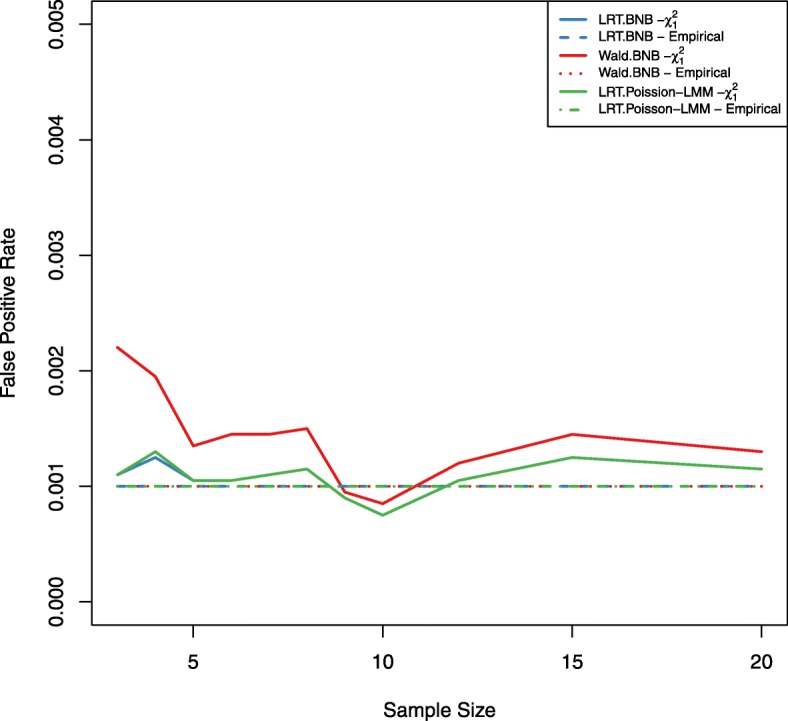
False positive rate plot for the TCGA breast cancer data set. Actual false positive rates were calculated for both the LRT and Wald tests under the BNB model and the LRT under the Poisson-LMM model at *μ*=14,*ϕ*=1, and 11 different sample sizes (range 3-20). The nominal false positive rate *α* is set at 0.001. The LRT and Wald tests under the BNB model and the LRT under the Poisson-LMM model were compared with the use of the empirical parametric test (dashed lines) and the Chi-square distribution (solid lines) under the null hypothesis

**Fig. 7:**
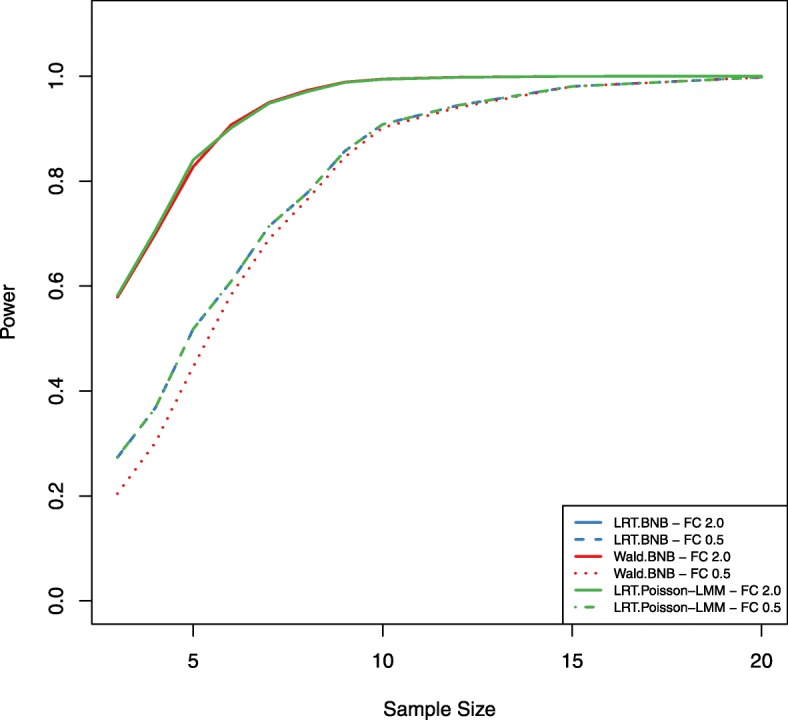
Power plot for the TCGA breast cancer data set. Power was calculated for both the LRT and Wald tests under the BNB model and the LRT under the Poisson-LMM model by using the empirical parametric test at *μ*=14,*ϕ*=1, and 11 different sample sizes (range 3-20). The nominal false positive rate *α* is set at 0.001. The detectable fold ratios of the mean expression levels between primary tumor and matched normal are set at 2 (solid lines) and 0.5 (dashed lines) under the alternative hypothesis

To study and demonstrate the proposed power estimation procedure in a real data application, we used the TCGA breast cancer data set as a pilot data set for designing a new study to detect differential expression using a paired design. The TCGA breast cancer data set BRCA (acquired in Feb. 2019 from FireBrowse) contains 1,212 tumor samples and 20,531 genes with non-zero counts. TMM method was used for normalization of all samples [22]. We chose the comparison between primary tumor samples and their matched normal samples (112 samples) for this case study. BNB model was fit to obtain parameter estimates for each gene. The estimated mean expression levels of matched normal samples are 14, 681, and 3,022 at the 20th, 50th, and 80th percentiles of all genes respectively. The estimated dispersion values are 0.07, 0.2, and 1 at the 20th, 50th, and 80th percentiles of all genes respectively. To demonstrate how to estimate power, we choose the mean expression level at the 20th percentile, the dispersion at the 80th percentile, and the detectable fold ratio of 2 and 0.5 between primary tumor and matched normal conditions to simulate test statistics under both the null and alternative hypotheses for 20,000 times. The LRT and Wald tests under the BNB model and the LRT under the Poisson-LMM model were compared with the use of the empirical parametric test as well as the Chi-square distribution under the null hypothesis at the nominal false positive rate of 0.001. Actual false positive rates using the Chi-square distribution at this *α* level are shown in Fig. 6. We observe that actual false positive rates are inflated at most sample sizes for the Wald test, which is consistent with the simulation results. Figure 7 shows power as a function of sample sizes at the mean expression level of 14 and the dispersion of 1 for a 2-fold up- or down-regulation of primary tumor compared to matched normal using the empirical parametric test for both tests. The LRT has better performance than the Wald test in general. To design a new RNA-Seq study with at least 80% power for the LRT, we will need *n*=5 samples per group to detect a 2-fold up-regulation or *n*=9 samples per group to detect a 2-fold down-regulation for genes at the mean expression level of 14 and the dispersion of 1. Results for all combinations of the mean expression level and the dispersion values are included in the additional file 1. The computation time for the BNB models and Poisson-LMM model are about 34 and 15 hours respectively on a standard windows laptop with Intel Core i7-6820HQ CPU at 2.70GHz and 32GB RAM. Two main factors for the used computation time in this case are the number (i.e. 20,000) of simulations and the number (i.e. 11) of different sample sizes.

## Discussion

Several studies discovered excessively inflated false positive rates for differential expression detection by using popular NB methods (i.e. edgeR, DESeq2) in RNA-Seq data analysis [23–26]. A reasonable explanation for this phenomena is that these methods mainly rely on biased asymptotic Chi-square distribution for inferences, which results in the failure in false positive rate control, especially in experiments with small sample sizes. In consistency with this phenomena, our published paper shows the downward bias of critical values when an asymptotic Chi-square distribution is applied for both the LRT and Wald tests under the NB model in power analysis for RNA-Seq differential expression studies [17]. In that paper, we provided a solution so that false positive rates can be controlled at the nominal level with the use of the empirical parametric test for obtaining critical values of both tests. In this paper, we show that either the empirical parametric test or asymptotic Chi-square distribution can be used to obtain critical values for both the LRT under the BNB model and the LRT under the Poisson-LMM model. However the empirical parametric test has to be used for both the Wald test under the BNB model and the LRT under the NB-LMM model because the test statistics deviate from the asymptotic Chi-square distribution.

We observed that the LRT under the BNB model and the LRT under the Poisson-LMM model have similar performance under both the null and alternative hypotheses. The main reason of the equivalent performance of these two tests is that the employed BNB model is based on the Poisson-Gamma compound distribution, which is similar to fit the Poisson-LMM model. When testing *γ*>1 for paired designs, the Wald test of BNB is recommended since it has the highest power among all four tests. But when testing *γ*<1 for paired designs, either the LRT under the BNB model or the LRT under the Poisson-LMM model is recommended. This unbalanced effect on power for the Wald test is related to selected transformation functions *g*(.), which was also reported in the article by Rettiganti and Nagaraja [13]. We also notice that there are some negative values of LRT statistics under the NB-LMM model in our simulations. For these cases with negative values, the parameter space under the null hypothesis is not completely contained in the parameter space under the alternative hypothesis since the MLE estimates of the random effect under both hypotheses are not the same.

In almost all current studies that use a NB model for differential expression detection or power analysis, the dispersion parameter is assumed equal across conditions. We have proposed unequal dispersion parameters across conditions for power analysis [17]. Similarly, the proposed BNB model can be naturally extended to unequal dispersion parameters with both the LRT and Wald tests for paired designs. In addition, the proposed GLMM procedure can be applied to multiple factorial designs and allow multiple random effects. For these designs, the value or distribution of model parameters need to be pre-specified so that the procedure can simulate data from a known GLMM model under both hypotheses. In addition, our proposed method can be used to estimate power at the data set level, where the distribution under the null hypothesis of the LRT or Wald test can be simulated for groups of genes with similar expression profile to maintain a proper false positive rate control.

## Conclusions

Many methods on sample size calculation and power estimation have already been proposed for RNA-Seq data over last decade. However nearly all of them were developed for experiments with independent measurements. To overcome this limitation in current methods, we provide a framework for power analysis of RNA-Seq experiments with correlated measurements (e.g. repeated samples, time course, etc.). Our simulation based procedures provide proper control of false positive rate, and our novel GLMM procedure can be used for complex designs by allowing diverse correlation structures with both random effects and random residual errors.

## Supplementary information

**Additional file 1** This pdf file contains all supplementary figures referenced in results section.

**Additional file 2** This text file contains all R functions used in simulations and the TCGA breast cancer data analysis.

**Additional file 3** This R markdown file contains R code and results for the TCGA breast cancer data analysis.

## Data Availability

The TCGA breast cancer data set BRCA used in the application section is from publicly available repositories (FireBrowse). R code for simulations and the TCGA data analysis is in the Additional files 2 and 3.

## Abbreviations

LRT: Likelihood ratio test
BNB: Bivariate negative binomial
MLE: Maximum likelihood estimation
GLMM: Generalized linear mixed effects model
PCER: Per comparison error rate
TCGA: The cancer genome atlas
