# Supplementary material for: Power analysis for RNA-Seq differential expression studies using generalized linear mixed effects models
Source: BMC Bioinformatics. 2020 May 19;21:198. doi: 10.1186/s12859-020-3541-7 (PMC7236949; doi:10.1186/s12859-020-3541-7)
Supplement: Supplementary file 1 — Additional file 1 This pdf file contains all supplementary figures referenced in results section. [file 12859_2020_3541_MOESM1_ESM.pdf]

Supplementary Figures:

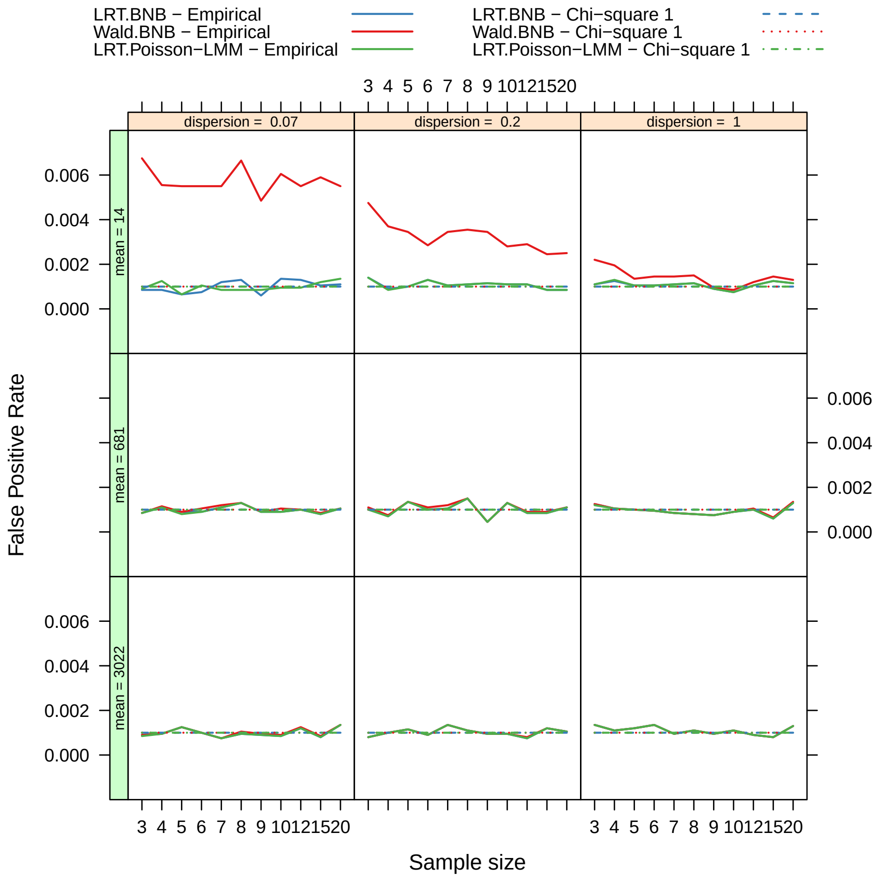

Figure 1 False positive rate plots for the TCGA breast cancer data set. Actual false positive rates were calculated for both the LRT and Wald tests under the BNB model and the LRT under the Poisson-LMM model at 9 different combinations of μ and ϕ, and 11 different sample sizes (range 3-20). The nominal false positive rate α is set at 0.001. The LRT and Wald tests under the BNB model and the LRT under the Poisson-LMM model were compared with the use of the empirical parametric test (dashed lines) and the Chi-square distribution (solid lines) under the null hypothesis.

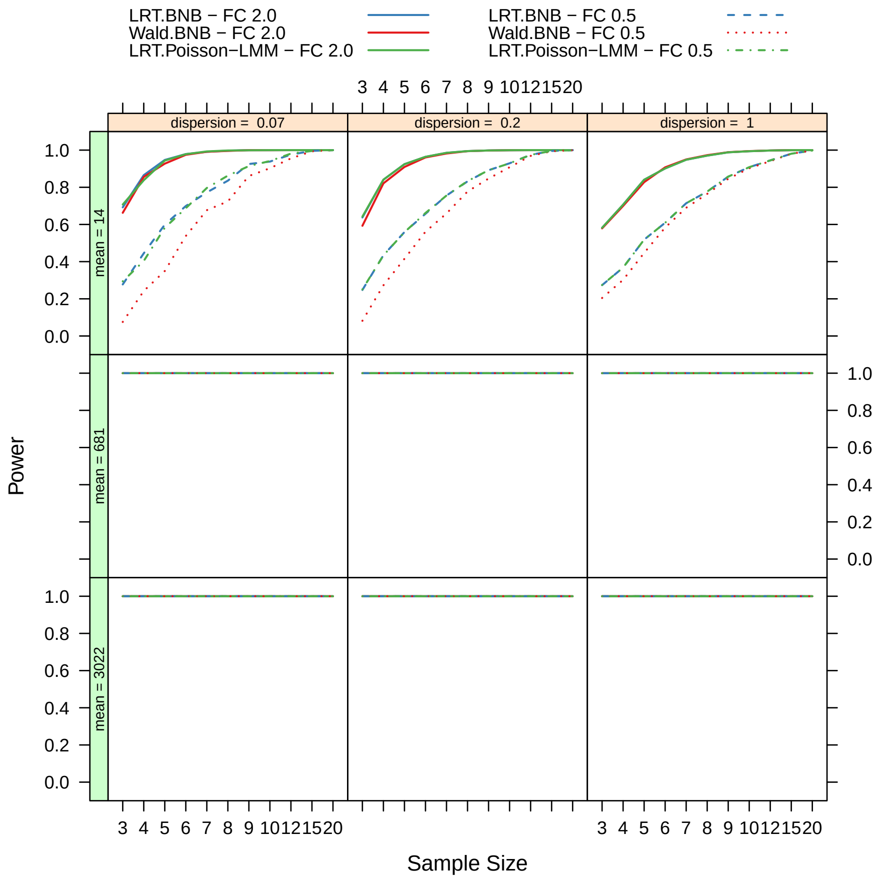

Figure 2 Power plots for the TCGA breast cancer data set. Power was calculated for both the LRT and Wald tests under the BNB model and the LRT under the Poisson-LMM model by using the empirical parametric test at 9 different combinations of μ and ϕ, and 11 different sample sizes (range 3-20). The nominal false positive rate α is set at 0.001. The detectable fold ratios of the mean expression levels between primary tumor and matched normal are set at 2 (solid lines) and 0.5 (dashed lines) under the alternative hypothesis.
