# Supplementary material for: Power analysis for RNA-Seq differential expression studies using generalized linear mixed effects models
Source: BMC Bioinformatics. 2020 May 19;21:198. doi: 10.1186/s12859-020-3541-7 (PMC7236949; doi:10.1186/s12859-020-3541-7)
Supplement: Supplementary file 3 — Additional file 3 This R markdown file contains R code and results for the TCGA breast cancer data analysis. [file 12859_2020_3541_MOESM3_ESM.html]

Power Analysis Using The TCGA Breast Cancer Data Set


# Power Analysis Using The TCGA Breast Cancer Data Set

#### Lianbo Yu

#### February 22, 2020

- **Data Simulation and Model Fitting**
- **Summary Statistics**
- **Plotting**

To start, source R functions and load the needed packages:

```
 source("R functions.R")
 library(lme4)
 library(maxLik)
 library(MASS) 
 library(RColorBrewer)
```

# **Data Simulation and Model Fitting**

Based on the specified model parameters, count data under both the null hypothesis and the alternative hypothesis are simulated by assuming a BNB model. LRT and Wald test under the BNB model and LRT under Poisson-LMM model are performed.

```
 ## set seed
 set.seed(1415)
 ## sample size (number of subjects)
 nvec <- c(3,4,5,6,7,8,9,10,12,15,20)
 ## 20%, 50%, and 80% expression of normal samples
 muvec.all <- c(14,681,3022)
 ## 20%, 50%, and 80% dispersion
 dispersionvec.all <- c(0.07,0.2,1)
 ## select 20% expression of normal samples
 muvec <- muvec.all[1] 
 ## select 80% dispersion of all samples
 thetavec <- 1/dispersionvec.all[3] 
 ## fold in primary samples vs. normals
 foldvec <- c(0.5, 1, 2) 
 ## expected false positive rate
 alphavec <- c(0.01, 0.005, 0.001)
 ## number of simulations
 T <- 20000
 
 ## arrays for test statistics
 array0.LRT.BNB <- array(0,dim=c(T,length(nvec),length(muvec),length(foldvec),length(thetavec)))
 array0.Wald.BNB <- array(0,dim=c(T,length(nvec),length(muvec),length(foldvec),length(thetavec)))
 array0.LRT.Poisson.Glmm <- array(0,dim=c(T,length(nvec),length(muvec),length(foldvec),length(thetavec)))

 f <- "TestResult.Rda"
 if (file.exists(f)) { load(f) } else 
 {
   for (l in 1:length(thetavec))
   {
     s1 <- thetavec[l]
     for (c in 1:length(foldvec))
     {
       for (k in 1:length(muvec))
       {  
         mu0 <- muvec[k]
         mu1 <- mu0*foldvec[c]
         for (j in 1:length(nvec))
         {  
           n <- nvec[j]
           for (i in 1:T)
           {
             data0 <- simubnb(n,mu0,mu1,s1)
             x <- data0$count[data0$group==0]
             y <- data0$count[data0$group==1]
             temp1 <- try(Test.BNB(x,y), silent=TRUE)
             if (!inherits(temp1,"try-error")) 
             {
               array0.LRT.BNB[i,j,k,c,l] <- temp1$lrt
               array0.Wald.BNB[i,j,k,c,l] <- temp1$stat2
             }
             temp2 <- try(Test.Poisson.Glmm(data0), silent=TRUE)
             if (!inherits(temp2,"try-error")) array0.LRT.Poisson.Glmm[i,j,k,c,l] <- temp2$lrt
           }
         }
       }
     }
   }
   save(array0.LRT.BNB,array0.Wald.BNB,array0.LRT.Poisson.Glmm,file=f)
 }
```

# **Summary Statistics**

False positive rates are calculated when the asymptotic Chi-square distribution is used to determine the critical values of the LRT and Wald tests.

```
 ## arrays for false positive rates
 fp.LRT.BNB <- array(0,dim=c(length(nvec),length(muvec),length(foldvec),
                             length(thetavec),length(alphavec)))
 fp.Wald.BNB <- array(0,dim=c(length(nvec),length(muvec),length(foldvec),
                              length(thetavec),length(alphavec)))
 fp.LRT.Poisson.Glmm <- array(0,dim=c(length(nvec),length(muvec),length(foldvec),
                                      length(thetavec),length(alphavec)))
 for (a in 1:length(alphavec))
 {
   for (l in 1:length(thetavec))
   {
     for (r in 1:length(foldvec))
     {
       for (k in 1:length(muvec))
       {  
         for (j in 1:length(nvec))
         {  
           fp.LRT.BNB[j,k,r,l,a] <- mean(array0.LRT.BNB[1:T,j,k,r,l] > 
                                           qchisq(1-alphavec[a],1),na.rm=TRUE)
           fp.Wald.BNB[j,k,r,l,a] <- mean(array0.Wald.BNB[1:T,j,k,r,l] > 
                                            qchisq(1-alphavec[a],1),na.rm=TRUE)
           fp.LRT.Poisson.Glmm[j,k,r,l,a] <- mean(array0.LRT.Poisson.Glmm[1:T,j,k,r,l] > 
                                                    qchisq(1-alphavec[a],1),na.rm=TRUE)
         }
       }
     }
   }
 }
```

To have a better false positive rate control than the usage of asypotic Chi-square distirbution under the null hypothesis, critical values are calculated by using the empirical parametric method.

```
 ## arrays for critival values
 critval.LRT.BNB <- array(0,dim=c(length(nvec),length(muvec),length(thetavec),length(alphavec)))
 critval.Wald.BNB <- array(0,dim=c(length(nvec),length(muvec),length(thetavec),length(alphavec)))
 critval.LRT.Poisson.Glmm <- array(0,dim=c(length(nvec),length(muvec),length(thetavec),length(alphavec)))
 for (a in 1:length(alphavec))
 {
    for (l in 1:length(thetavec))
    {  
       for (k in 1:length(muvec))
       {  
          for (j in 1:length(nvec))
          {  
             critval.LRT.BNB[j,k,l,a] <- 
               quantile(array0.LRT.BNB[1:T,j,k,2,l],1-alphavec[a],na.rm=TRUE)
             critval.Wald.BNB[j,k,l,a] <- 
               quantile(array0.Wald.BNB[1:T,j,k,2,l],1-alphavec[a],na.rm=TRUE)
             critval.LRT.Poisson.Glmm[j,k,l,a] <- 
               quantile(array0.LRT.Poisson.Glmm[1:T,j,k,2,l],1-alphavec[a],na.rm=TRUE)
          }
       }
    }
 }
```

Power is calculated under the alternative hypothesis by using the above critical values.

```
 ## arrays for power
 power.LRT.BNB <- array(0,dim=c(length(nvec),length(muvec),length(foldvec),
                                length(thetavec),length(alphavec)))
 power.Wald.BNB <- array(0,dim=c(length(nvec),length(muvec),length(foldvec),
                                 length(thetavec),length(alphavec)))
 power.LRT.Poisson.Glmm <- array(0,dim=c(length(nvec),length(muvec),length(foldvec),
                                         length(thetavec),length(alphavec)))
  for (a in 1:length(alphavec))
 {
    for (l in 1:length(thetavec))
    {
       for (r in 1:length(foldvec))
       {
          for (k in 1:length(muvec))
          {  
             for (j in 1:length(nvec))
             {  
                power.LRT.BNB[j,k,r,l,a] <- mean(array0.LRT.BNB[1:T,j,k,r,l] > 
                                                   critval.LRT.BNB[j,k,l,a],na.rm=TRUE)
                power.Wald.BNB[j,k,r,l,a] <- mean(array0.Wald.BNB[1:T,j,k,r,l] > 
                                                    critval.Wald.BNB[j,k,l,a],na.rm=TRUE)
                power.LRT.Poisson.Glmm[j,k,r,l,a] <- mean(array0.LRT.Poisson.Glmm[1:T,j,k,r,l] > 
                                                          critval.LRT.Poisson.Glmm[j,k,l,a],na.rm=TRUE)
             }
          }
       }
    }
 }
```

# **Plotting**

False positive rate is plotted against sample size.

```
 plot(nvec,fp.LRT.BNB[,1,2,1,3],xlim=c(3,20),ylim=c(0,0.01),type="l",
      xlab="Sample Size",ylab="False Positive Rate",col=brewer.pal(6,"Set1")[2],lwd=2)
 points(nvec,fp.Wald.BNB[,1,2,1,3],col=brewer.pal(6,"Set1")[1],type="l",lwd=2) 
 points(nvec,power.LRT.BNB[,1,2,1,3],col=brewer.pal(6,"Set1")[2],type="l",lty=2,lwd=2) 
 points(nvec,power.Wald.BNB[,1,2,1,3],col=brewer.pal(6,"Set1")[1],type="l",lty=3,lwd=2) 
 points(nvec,fp.LRT.Poisson.Glmm[,1,2,1,3],col=brewer.pal(6,"Set1")[3],type="l",lwd=2) 
 points(nvec,power.LRT.Poisson.Glmm[,1,2,1,3],col=brewer.pal(6,"Set1")[3],type="l",lty=4,lwd=2) 
 legend("topright",legend=c(expression({"LRT.BNB -"*chi[1]^2}),"LRT.BNB - Empirical",
                            expression({"Wald.BNB -"*chi[1]^2}),"Wald.BNB - Empirical",
                            expression({"LRT.Poission-LMM -"*chi[1]^2}),"LRT.Poisson-LMM - Empirical"),
        lty=c(1,2,1,3,1,4),lwd=2,col=c(brewer.pal(3,"Set1")[2],brewer.pal(3,"Set1")[2],
                                   brewer.pal(3,"Set1")[1],brewer.pal(3,"Set1")[1],
                                   brewer.pal(3,"Set1")[3],brewer.pal(3,"Set1")[3]),cex=0.7)
```

Power is plotted against sample size.

```
 plot(nvec,power.LRT.BNB[,1,3,1,3],xlim=c(3,20),ylim=c(0,1.1),type="l",
      xlab="Sample Size",ylab="Power",col=brewer.pal(6,"Set1")[2],lwd=2)
 points(nvec,power.Wald.BNB[,1,3,1,3],col=brewer.pal(6,"Set1")[1],type="l",lwd=2) 
 points(nvec,power.LRT.BNB[,1,1,1,3],col=brewer.pal(6,"Set1")[2],type="l",lty=2,lwd=2) 
 points(nvec,power.Wald.BNB[,1,1,1,3],col=brewer.pal(6,"Set1")[1],type="l",lty=3,lwd=2) 
 points(nvec,power.LRT.Poisson.Glmm[,1,3,1,3],col=brewer.pal(6,"Set1")[3],type="l",lwd=2) 
 points(nvec,power.LRT.Poisson.Glmm[,1,1,1,3],col=brewer.pal(6,"Set1")[3],type="l",lty=4,lwd=2) 
 legend("bottomright",legend=c("LRT.BNB - FC 2.0","LRT.BNB - FC 0.5",
                               "Wald.BNB - FC 2.0","Wald.BNB - FC 0.5",
                               "LRT.Poisson-LMM - FC 2.0","LRT.Poisson-LMM - FC 0.5"),
        lty=c(1,2,1,3,1,4),lwd=2,col=c(brewer.pal(3,"Set1")[2],brewer.pal(3,"Set1")[2],
                                       brewer.pal(3,"Set1")[1],brewer.pal(3,"Set1")[1],
                                       brewer.pal(3,"Set1")[3],brewer.pal(3,"Set1")[3]),cex=0.7)
```
